# Noninterference Revealing of “Layered to Layered” Zinc Storage Mechanism of δ‐MnO_2_ toward Neutral Zn–Mn Batteries with Superior Performance

**DOI:** 10.1002/advs.201902795

**Published:** 2020-01-16

**Authors:** Yuqi Jiang, Deliang Ba, Yuanyuan Li, Jinping Liu

**Affiliations:** ^1^ State Key Laboratory of Advanced Technology for Materials Synthesis and Processing and School of Chemistry Chemical Engineering and Life Science Wuhan University of Technology Wuhan Hubei 430070 P. R. China; ^2^ School of Optical and Electronic Information Huazhong University of Science and Technology Wuhan Hubei 430074 P. R. China; ^3^ State Center for International Cooperation on Designer Low‐carbon & Environmental Materials and School of Materials Science and Engineering Zhengzhou University Zhengzhou Henan 450001 P. R. China

**Keywords:** aqueous neutral Zn–MnO_2_ batteries, δ‐MnO_2_, quasi‐solid‐state batteries, zinc storage mechanism, Zn–Mn batteries

## Abstract

MnO_2_ is one of the most studied cathodes for aqueous neutral zinc‐ion batteries. However, the diverse reported crystal structures of MnO_2_ compared to δ‐MnO_2_ inevitably suffer a structural phase transition from tunneled to layered Zn‐buserite during the initial cycles, which is not as kinetically direct as the conventional intercalation electrochemistry in layered materials and thus poses great challenges to the performance and multifunctionality of devices. Here, a binder‐free δ‐MnO_2_ cathode is designed and a favorable “layered to layered” Zn^2+^ storage mechanism is revealed systematically using such a “noninterferencing” electrode platform in combination with ab initio calculation. A flexible quasi‐solid‐state Zn–Mn battery with an electrodeposited flexible Zn anode is further assembled, exhibiting high energy density (35.11 mWh cm^−3^; 432.05 Wh kg^−1^), high power density (676.92 mW cm^−3^; 8.33 kW kg^−1^), extremely low self‐discharge rate, and ultralong stability up to 10 000 cycles. Even with a relatively high δ‐MnO_2_ mass loading of 5 mg cm^−2^, significant energy and power densities are still achieved. The device also works well over a broad temperature range (0–40 °C) and can efficiently power different types of small electronics. This work provides an opportunity to develop high‐performance multivalent‐ion batteries via the design of a kinetically favorable host structure.

## Graphical abstract

A “layered to layered” mechanism for zinc storage in δ‐MnO_2_ is revealed based on a binder‐/additive‐free “noninterferencing” electrode platform. A δ‐MnO_2_‐based flexible quasi‐solid‐state zinc–manganese battery is further designed, which achieves high energy density, high power density, and outstanding cycling stability up to 10 000 times and exhibits good mechanical properties and a low self‐discharge rate.

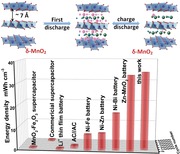

## 1 Introduction

Aqueous zinc‐ion batteries (ZIBs) have attracted extensive attention due to the low equilibrium potential of Zn, their superior energy densities, lower cost, and environmental friendliness compared to typical aqueous alkali‐metal‐ion batteries.^1^, ^2^, ^3^, ^4^, ^5^, ^6^ In recent years, many efforts have been made to develop aqueous ZIBs with diverse cathodes, such as Zn–MnO_2_ batteries,^7^, ^8^, ^9^ Zn–Ag_2_O batteries,^10^ Zn–NiOOH batteries,^11^, ^12^ Zn–V_2_O_5_ batteries,^13^, ^14^ Zn–Co_3_O_4,_ 15 and Zn–Na_3_V_2_(PO_4_)_3_ batteries,^16^, ^17^ etc. Among them, aqueous Zn–MnO_2_ batteries are quite promising due to their relatively high capacity with Zn^2+^ insertion, nontoxicity, and low cost associated with the MnO_2_ cathode.^1^, ^6^ To date, primary Zn–MnO_2_ batteries have been commercialized and alkaline aqueous rechargeable Zn–MnO_2_ batteries have been developed. Nevertheless, issues remain, such as Zn anode corrosion in alkaline electrolytes and irreversible reduction of MnO_2_ with deep discharging. Additionally, alkaline electrolytes are environmentally unfriendly and difficult to recycle, which greatly restricts the practical application of alkaline secondary Zn–MnO_2_ batteries.^18^, ^19^ In 2012, a rechargeable Zn–MnO_2_ battery based on neutral electrolytes such mild aqueous ZnSO_4_ was invented to address the above issues, which exhibited high safety, a high capacity of 210 mAh g^−1^ and a good recharge ability at a 100% depth of discharge.^9^ Encouraged by this advance, in the past few years, many attempts have been made to uncover the Zn^2+^ ion storage mechanism in various phases of MnO_2_ and to develop advanced aqueous neutral Zn–MnO_2_ batteries (ANZMBs).

**Figure 1:**
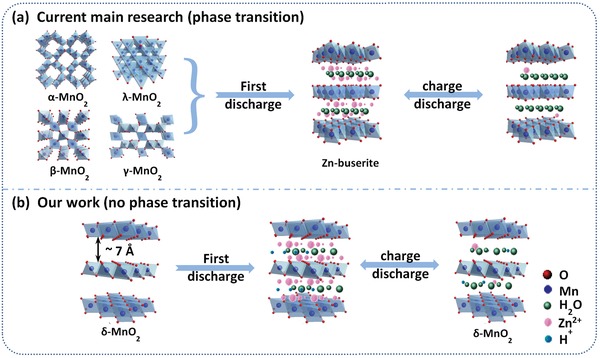
Schematic illustration of different crystal structures of MnO_2_ and the zinc storage mechanisms.

Until now, a wide spectrum of MnO_2_ cathodes has been investigated for ANZMBs, and most of them possess tunnel‐like crystal structures. These include α‐MnO_2_ (2*2 tunnels), β‐MnO_2_ (1*1 tunnels), γ‐MnO_2_ (1*2 and 1*1 tunnels), λ‐MnO_2_ (1*3 tunnels), etc. (**Figure** **1**a).^7^, ^9^, ^20^, ^21^ Such tunnel structures provide accumulation sites for Zn^2+^, which makes it possible to achieve excellent Zn^2+^ storage capability. However, the structural changes for different MnO_2_ phases upon Zn uptake and their reversible reaction mechanisms have been controversial for a long time. Several previous studies reported that a conversion reaction process occurred during Zn^2+^ uptake, while others considered the process as an intercalation mechanism (e.g., formation of ZnMn_2_O_4_).^9^, ^22^, ^23^, ^24^ Most recently, Cheng et al.^20^, ^25^ analyzed previous works in detail and revealed a versatile mechanism; in general, the tunnel‐structured MnO_2_ is transferred to layered zinc‐buserite during the first cycle, allowing the intercalation of zinc cations in the resulting layered structure in subsequent cycles. Even though the first‐cycle phase transition is energetically favorable, the conversion is not as kinetically direct as in conventional intercalation electrochemistry, and may impact the electrode stability.

Different from α‐, β‐, γ‐, and λ‐MnO_2_, δ‐MnO_2_ intrinsically has a typical layered structure with the interlayer spacing of ≈7 Å (Figure 1b). Such a structure has been demonstrated with high Li ion mobility and little structural rearrangement upon cycling in Li ion batteries.^26^, ^27^, ^28^ Similarly, the large‐interspacing architecture may allow reversible and abundant Zn^2+^ ion intercalation and de‐intercalation.^29^ The low proton adsorption energy of δ‐MnO_2_ structure[qv: 28b] may also help to reduce the proton co‐insertion effect. Consequently, an energetically and kinetically favorable “layered to layered” structure transformation is expected for Zn^2+^ storage with δ‐MnO_2_. Nevertheless, to our knowledge, only a few reports have been reported on using δ‐MnO_2_ in ANZMBs, and in particular, the expected structural transformation mechanism is still disputed^30^ and needs clarification via eliminating the interference from binders/additives in traditional electrodes.

On the other hand, although mild neutral electrolytes can be used in ANZMBs, electrolyte leakage problems still exist. Utilization of (quasi‐)solid‐state electrolytes in ANZMBs can address this issue and simplify device packaging.^31^ Furthermore, the development of emerging flexible and wearable electronics has greatly stimulated the design of flexible electrochemical energy storage devices.^32^, ^33^ In this regard, the integration of (quasi‐)solid‐state electrolytes would further enhance the flexibility and stability of such devices. Despite these advantages, δ‐MnO_2_ has rarely been employed to construct flexible ANZMBs. In addition, simultaneously achieving high energy/power densities as well as ultralong cycling durability for flexible ANZMBs remains challenging.

Here, we report a δ‐MnO_2_‐based flexible ANZMB, in which both the δ‐MnO_2_ cathode and Zn anode are directly grown on a flexible current collector using a simple electrodeposition method. Such an “additive/binder‐free” electrode architecture allows us to precisely understand the charge storage mechanism. As expected, a “layered to layered” structural evolution process of δ‐MnO_2_ during charge and discharge is uncovered through systematic analysis of the crystal structure, component, morphology and ionic valence. The ab initio molecular dynamics (AIMD) simulation also supports the revealed mechanism. No phase transition effectively maintains the electrode stability and improves the cycle life. With the deposited Zn thin film (instead of traditional heavy and nonflexible Zn foil) as an anode and poly(vinyl alcohol) (PVA)–ZnSO_4_‐based gel as an electrolyte, a highly flexible quasi‐solid‐state ZMB is further developed, which exhibits an average discharge voltage of ≈1.5 V, a good rate capability and an outstanding cycling stability up to 10 000 times. The device also delivers high energy density and power density based on the total mass of cathode and anode active materials or based on the entire device volume, which is better than recently reported aqueous and flexible devices. Interestingly, our device has a very low self‐discharge and wide temperature operation capability (0–40 °C), which have not yet been demonstrated in flexible ANZMBs.

## 2 Results and Discussion

### 2.1 Characterizations of the δ‐MnO_2_ Cathode

**Figure 2:**
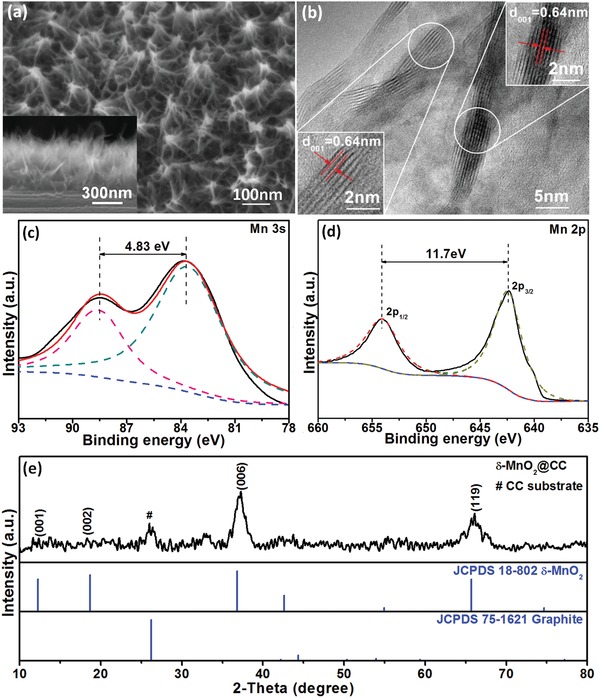
Structural and morphological characterizations of δ‐MnO_2_: a) SEM and b) HRTEM images. c) XPS core level Mn 3s spectrum and d) Mn 2p spectrum. e) XRD pattern.

The layered δ‐MnO_2_ cathode was synthesized by facile, one‐step electrochemical deposition, which gives rise to a homogenous self‐supporting array film structure. As shown by scanning electron microscopy (SEM) images in **Figure** **2**a and Figure S1 (Supporting Information), the as‐grown film is composed of numerous ultrathin nanoflakes on carbon cloth (CC) fibers. The tiny flakes are highly cross‐linked with each other, forming an array film thickness of ≈510 nm (inset in Figure 2a). A high‐resolution transmission electron microscopy (HRTEM) image is further shown in Figure 2b, which clearly shows that δ‐MnO_2_ nanoflakes are well crystallized with thicknesses of merely 3–5 nm. The flakes have a unique layered structure with a lattice spacing of ≈0.64 nm, corresponding to the (001) plane. To clarify the valence state of Mn, X‐ray photoelectron spectroscopy (XPS) measurements were performed. The full XPS spectrum is shown in Figure S2 (Supporting Information), which exactly reveals the presence of Mn and O elements. Additionally, the splitting width of 4.83 eV of the Mn 3s spectrum indicates that Mn^4+^ is the dominant state in the Mn oxide (Figure 2c). For the Mn 2p spectrum (Figure 2d), the two peaks at binding energies of 642.38 and 654.08 eV with a spin‐energy separation of 11.7 eV correspond to Mn 2p_3/2_ and Mn 2p_1/2_ in Mn(IV)O_2_, respectively.^20^ This component of the film was also supported by X‐ray diffraction (XRD) results. As shown in Figure 2e, all diffraction peaks can be assigned to δ‐MnO_2_ (JCPDS card No. 18–802), except the signal near 26° from the current collector. The relatively high intensity of (006) peak indicates a preferred orientation of the nanoflake film.

### 2.2 Revealing Zn^2+^ Storage Mechanism in Noninterferencing δ‐MnO_2_ Electrode

**Figure 3:**
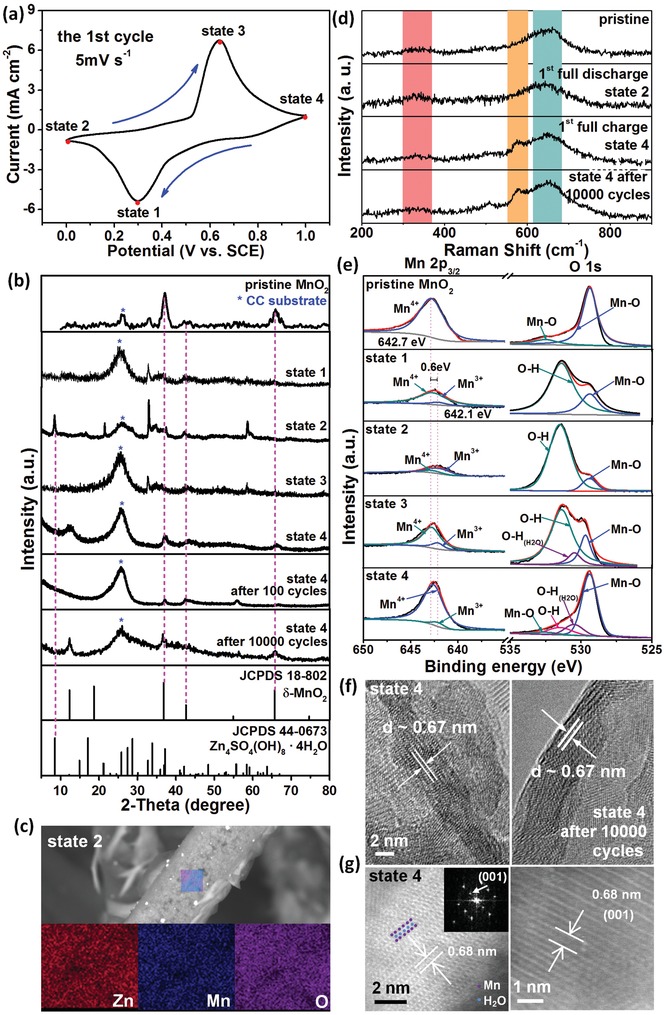
a) First CV curve of the δ‐MnO_2_ cathode. b) XRD patterns of δ‐MnO_2_ at different states. c) SEM and elemental mapping toward state 2. d) Raman spectra of δ‐MnO_2_ at different states. e) Core level Mn 2p and O 1s spectra at different states. f) HRTEM images of state 4 and state 4 after 10 000 cycling. g) The HAADF image of state 4 after cycles revealing the layered atomic structure.

To investigate the electrochemical properties of δ‐MnO_2_ cathodes for Zn^2+^ storage, a mixed electrolyte of ZnSO_4_ and MnSO_4_ was employed. A small amount of Mn^2+^ was added to the electrolyte to change the dissolution equilibrium and inhibit the disproportionation reaction of Mn^3+^.^34^ Additionally, it has been reported that Mn^2+^ might improve the Zn plating/stripping efficiency.^34^ As displayed in **Figure** **3**a, the first‐cycle CV profile of the δ‐MnO_2_ electrode exhibits well‐defined redox peaks.

To understand the Zn^2+^ storage mechanism, the electrode was charged and discharged to different states, for which the phase, crystal structure, morphology, etc. were systematically analyzed. In Figure 3b, the XRD evolution results of the δ‐MnO_2_ electrode are given first. It is apparent that with the electrode being gradually discharged (state 1, state 2), two typical peaks (≈36.8° and 42.6°) from δ‐MnO_2_ can still be observed, preliminarily indicating that Zn intercalation does not change the phase of MnO_2_. The other diffraction peaks at 8.52°, 21.085°, and 33.849° gradually appear and correspond well to Zn_4_SO_4_(OH)_6_•4H_2_O (JCPDS card No. 44–0673). This phenomenon was also found in other phased MnO_2_ cathodes and was ascribed to H^+^ co‐insertion, with which the locally generated OH^−^ was assembled on the electrode surface and promoted the deposition of Zn salt.^23^, ^34^, ^35^ The SEM image in Figure S3 (Supporting Information) confirms the presence of large pieces of Zn_4_SO_4_(OH)_6_·4H_2_O attached to the surface of the δ‐MnO_2_ electrode. Energy‐dispersive X‐ray spectroscopy (EDS) analysis toward state 2 (Figure 3c) shows that where Zn salt is not present, there are still obvious Zn signals together with Mn and O in the discharge product, indicative of successful Zn insertion into MnO_2_. When the electrode was charged to state 3 and state 4, the XRD peaks from the Zn salt decrease and finally almost disappear with other peaks indexed well to δ‐MnO_2_. In addition, even after 100 and 10 000 times of cycling, δ‐MnO_2_ is still the dominant phase of the electrode despite that the peak intensities are decreased as compared to the initial state. Raman spectra (Figure 3d) were further utilized to probe the structural evolution upon Zn^2+^ insertion/extraction. The bands at 650 cm^−1^ and 575–585 cm^−1^ are characteristic of the layered δ‐MnO_2_.^36^, ^37^ During repeated cycling, the peak of 650 cm^−1^ is almost kept unchanged, indicative of the structural stability of δ‐MnO_2_. The increased intensity of the band at 575–585 cm^−1^ is due to the enhanced Mn—O stretching in the basal plane of the MnO_6_ sheet.^37^ Upon discharging and charging, the emergence and disappearance of the overlapped broad peaks of 325/375 cm^−1^ associated with Zn—O stretching confirms the reversible zinc storage process. All the above results clearly imply reversible Zn^2+^ insertion/de‐insertion and a “layered to layered” structural evolution mechanism with no obvious phase transition.

To further confirm the mechanism, an XPS survey was conducted for different states and compared to the pristine δ‐MnO_2_ electrode (Figures S2 and S4, Supporting Information). In general, Zn 2p signals are detected in the discharged states (state 1 and state 2) and significantly vanish upon charging (state 3 and state 4). Specifically, core‐level Mn 2p_3/2_ and O 1s spectra were analyzed (deconvoluted) to probe the valence state change of elemental Mn (Figure 3e). In the pristine state, the peak located at 642.7 eV of Mn 2p_3/2_ corresponds to a Mn^4+^ oxidation state. With the δ‐MnO_2_ electrode gradually discharged (state 1 and state 2), a new peak arises at 642.1 eV that is characteristic of Mn^3+^,^38^ and the peak of Mn^4+^ diminishes greatly, implying the reduction of Mn^4+^ to Mn^3+^ caused by Zn^2+^ insertion into the δ‐MnO_2_ layer. In the O1s spectra of state 1 and state 2, different from Mn—O peaks at 529.38 and 532.48 eV for the pristine electrode, a distinct peak at 531.6 eV is detected and gradually increased, which can be indexed to the O—H bond;^23^ this peak is believed to be associated with the basic zinc sulfate generated on the electrode surface, as discussed for the XRD analysis. Furthermore, after the electrode was charged (state 3 and state 4), the major peak of Mn^3+^ at 642.1 eV significantly decreased, whereas that of Mn^4+^ was dominant in the Mn 2p_3/2_ spectrum. The deconvoluted peaks of O 1s at state 4 are also nearly consistent with the pristine δ‐MnO_2_ electrode, indicative of Zn^2+^ de‐insertion from the interlayer (the weak O—H bonds at 531.6 and 530.5 eV are due to the remaining attached basic zinc sulfate and crystalline water).

SEM image of the electrode at state 4 after 10 000 cycles demonstrates that the nanoflake structure can be well maintained (Figure S5, Supporting Information). HRTEM images of state 4 and state 4 after 10 000 cycles (Figure 3f and Figure S6, Supporting Information) further confirm a preserved layered structure with an interlayer spacing of ≈0.67 nm. High‐angle‐annular‐dark‐field scanning transmission electron microscopy (HAADF‐STEM) images of state 4 (Figure 3g) show clearer layered structure, where the Mn and H_2_O slabs can be observed due to their different contrasts. Compared to the pristine δ‐MnO_2_, the interspacing value is slightly larger, which is indicative of the expansion effect of Zn^2+^ insertion/de‐insertion. The diffusion coefficient of Zn^2+^ (*D*_Zn2+_) in δ‐MnO_2_ was estimated using the galvanostatic intermittent titration technique (GITT) technology. As shown in Figure S7 (Supporting Information), the average *D*_Zn2+_ values during charging and discharging plateaus are ≈3.145 × 10^−11^ and 2.21 × 10^−11^ cm^2^ s^−1^, respectively. The values are larger than those in spinel structure electrode,^25^ indicating that Zn^2+^ diffusion is indeed facilitated in the interlayers of δ‐MnO_2_.

**Figure 4:**
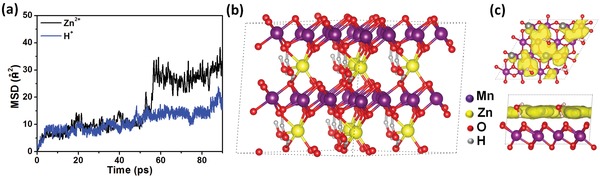
The ab initio simulation calculation. a) MSD of Zn^2+^ and H^+^ in layered δ‐MnO_2_. b) The calculated structure of state 2. c) Projections of diffusion trajectories of Zn^2+^ from the top view and the side view.

Besides the experimental evidences, the Zn^2+^ insertion mechanism and transport behavior in δ‐MnO_2_ (with H^+^ co‐insertion) can be predicted using AIMD method.^39^ The changes in the mean square displacements (MSD) of Zn^2+^ and H^+^ in layered δ‐MnO_2_ are illustrated in **Figure** **4**a. It is clear that, driven by H_2_O molecules, Zn^2+^ can transport smoothly in the interlayers of δ‐MnO_2_ and the layered structure with large spacing is energetically and dynamically stabilized. The intercalated structure is given in Figure 4b, in which Zn^2+^ and H^+^ co‐exist stably between the layers with the help of H_2_O molecules. The Zn^2+^ ion trajectories are displayed in Figure 4c. As can be seen, Zn^2+^ can realize long‐range transportation in Mn—O hexahedron layers, and the layered structure units are maintained over the AIMD process.

### 2.3 Electrochemical Performance of δ‐MnO_2_ Cathode

We next investigate electrochemical performance. The effect of loading mass on the performance of δ‐MnO_2_ electrode was investigated (Figure S8a,b, Supporting Information) and we chose the electrode with relatively large loading mass, high specific capacity and rate performance for next experiments. Figure S8c (Supporting Information) illustrates the CV curves of the δ‐MnO_2_ electrode, in which symmetric redox peaks can be seen at different scan rates ranging from 1 to 50 mV s^−1^. At 1 mV s^−1^, the cathodic peaks at 0.35 V are well indexed to the reduction of Mn^4+^ to Mn^3+^ with the Zn^2+^ insertion into layered δ‐MnO_2_, while the anodic peak at ≈0.6 V corresponds to the oxidation of Mn^3+^ to Mn^4+^ associated with Zn^2+^ de‐insertion. ^20^ In accordance with the CVs, the galvanostatic charge–discharge (GCD) curves for δ‐MnO_2_ display obvious plateaus at various current densities (Figure S8d, Supporting Information).

The rate performance and cycling stability are further shown in Figure S8e,f (Supporting Information). It is worth noting that the δ‐MnO_2_ cathode exhibits almost 100% capacity retention after 6000 cycles at 5 mA cm^−2^. The high coulombic efficiency (≈99.5%) is indicative of the good electrochemical reversibility of Zn^2+^ insertion/de‐insertion. The cycling performance of our δ‐MnO_2_ array film is superior to those of many previous MnO_2_ electrodes, such as MnO_2_@PEDOT (83.7% retention after 300 cycles),^31^ ZnMn_2_O_4_ (94% retention after 500 cycles),^25^ MnO_2_/graphene scrolls (94% retention after 3000 cycles),^40^ and polyaniline‐intercalated MnO_2_ (87% retention after 5000 cycles).^29^ The excellent cyclic stability should benefit from the large interlayer of the unique lamellar structured δ‐MnO_2_, which provides a direct channel for Zn^2+^ transport and thus avoids the destruction of the host structure during repeated cycling.

### 2.4 Design and Performance of Zn–Mn Full Cell

**Figure 5:**
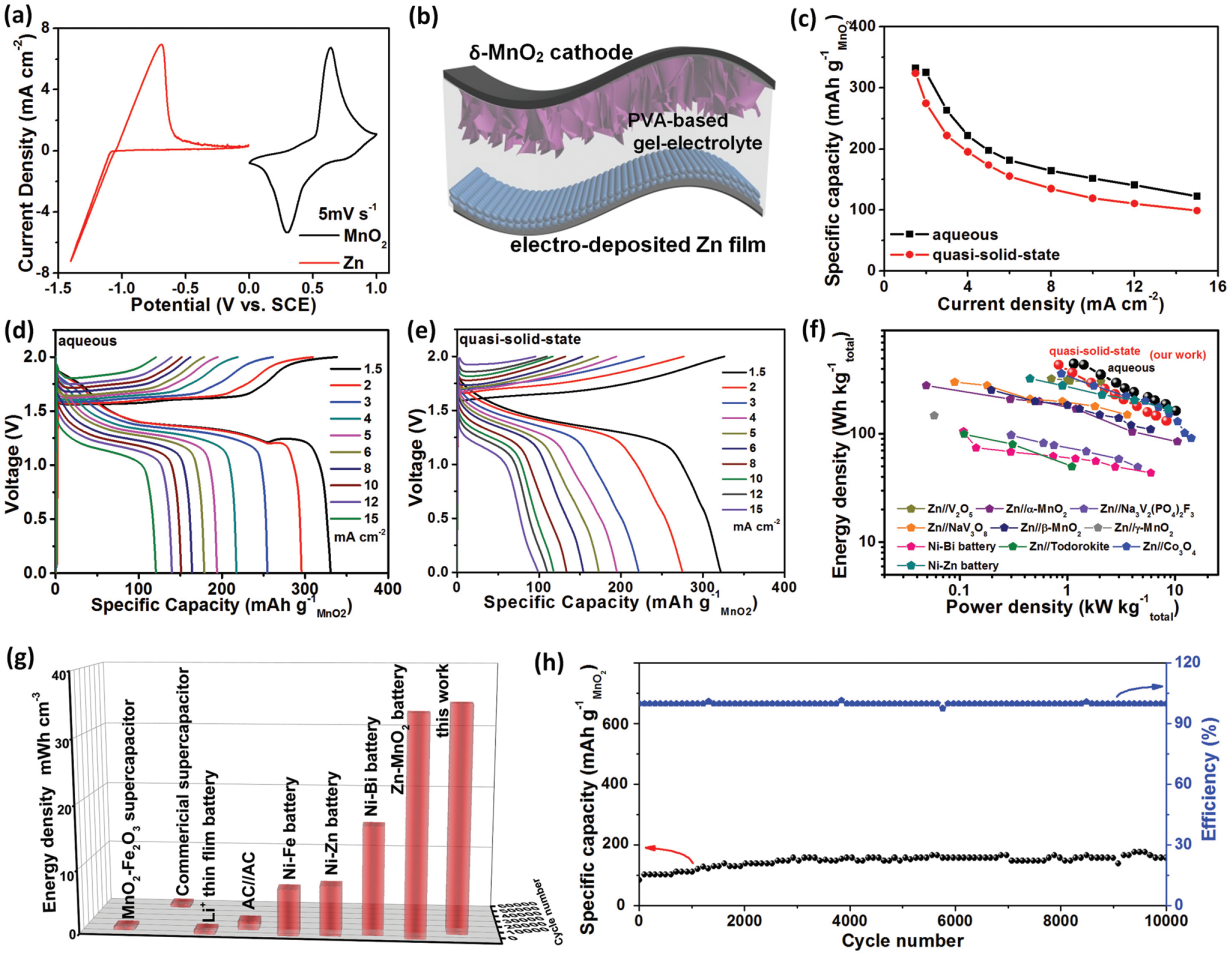
Electrochemical performance of ZMBs: a) CV curves of δ‐MnO_2_ cathode and Zn anode performed in three‐electrode cell at 5 mV s^−1^. b) Schematic diagram of the quasi‐solid‐state flexible ZMB. c) Specific capacities of ZMBs with aqueous and quasi‐solid‐state electrolytes. d) Charge–discharge profiles of ZMB with aqueous electrolyte. e) Charge–discharge profiles of ZMB with quasi‐solid‐state electrolyte. f) Ragone plot of flexible ZMB based on the total mass of active materials. g) Comparison of volumetric energy density and cycling stability of our flexible device and those presented in previous works. h) Cycling performance at 5 mA cm^−2^ of our flexible ZMB.

To further evaluate the potential of the layered δ‐MnO_2_ film cathode for ANZMB application, a Zn anode was synthesized on CC using a cathodic electrodeposition method. SEM images (Figure S9a and the inset, Supporting Information) show that after deposition, homogeneous Zn metal film is tightly adhered to the current collector with a thickness of ≈270 nm, which is much thinner and more flexible than traditional Zn foil anodes. The thinner Zn film is good for improving the flexibility and energy density of the device. The XRD pattern (Figure S9b, Supporting Information) confirms the generation of hexagonal zinc (JCPDS card 87–0713). **Figure** **5**a illustrates the CV curves of both the as‐prepared Zn anode and δ‐MnO_2_ cathode at 5 mV s^−1^. The calculated stored charges of the two electrodes are found to match well; accordingly, a possible broad voltage window of ≈2.0 V versus Zn^2+^/Zn for the ANZMB device is expected. The good matching of Zn and δ‐MnO_2_ electrodes is further evidenced by the CVs and GCD profiles of the aqueous full cell, which exhibit characteristic redox peaks and plateaus, respectively, with coulombic efficiencies approaching 100% (Figure S10, Supporting Information).

For practical application, aqueous electrolytes have the risk of leakage, and with the aim to develop flexible and light‐weight power sources with superior properties, a quasi‐solid‐state ZMB device using a PVA–ZnSO_4_‐based gel electrolyte (Figure 5b) is designed and evaluated. It is worth noting that there are few reports on using δ‐MnO_2_ in flexible solid‐state ZMBs. Interestingly, the rate performance of our gel electrolyte‐based device is not much different from that in the liquid state, as compared in Figure 5c. Although the specific capacities of the quasi‐solid‐state ZMB are slightly smaller than those obtained with the liquid electrolyte (due to relatively poor ionic conductivity of the gel electrolyte and the imperfect interfacial contact between electrolyte/material; the conductivity and contact issue should also account for the relatively higher voltage polarization, as will be observed later), high capacities can still be achieved within a wide range of current density. In particular, for the quasi‐solid‐state device, a capacity of ≈100 mAh g^−1^_MnO2_ can be retained by increasing the current 10 times to 15 mA cm^−2^, demonstrating its fast‐charging capability. The GCD curves for the ZMB devices with the gel electrolyte and liquid electrolyte are further presented in Figure 5d,e, respectively. As is shown, in the liquid electrolyte, the device can achieve a quite high discharge capacity of 330.67 mAh g^−1^_MnO2_ at 1.5 mA cm^−2^, outperforming most of the recent nonlayered MnO_2_‐based ZMB batteries and other aqueous rechargeable batteries.^7^, ^8^, ^24^, ^41^ With the PVA–ZnSO_4_‐based electrolyte, the device also exhibits well‐defined CV profiles (Figure S11, Supporting Information) and featured GCD curves that are characteristic of good plateaus with voltage hysteresis not apparently increased. Specifically, a remarkable capacity of 324.04 mAh g^−1^_MnO2_ at a current density of 1.5 mA cm^−2^ is obtained, which surpasses most recently reported quasi‐solid‐state Zn–MnO_2_ batteries.^31^, ^32^, ^41^ Similar to the device with liquid electrolyte, the quasi‐solid‐state device exhibits high coulombic efficiencies of 98–100% at various current densities.

To further highlight the superiority of our quasi‐solid‐sate ZMB, a Ragone plot reflecting the relationship between energy density and power density is displayed in Figure 5f and is compared to other electrochemical energy storage devices in the literature. Considering the total mass of the cathode and anode materials, our quasi‐solid‐state ZMB delivers a maximum gravimetric energy density of 432.05 Wh kg^−1^ and a maximum power density of 8.33 kW kg^−1^. The energy density value is much better than those of typical previous energy storage systems, such as Ni//Zn batteries (323.3 Wh kg^−1^),^42^ Ni//Bi batteries (105 Wh kg^−1^),^43^ Zn//V_2_O_5_ batteries (144 Wh kg^−1^, 274 Wh kg^−1^),^13^, ^14^ Zn//Co_3_O_4_ batteries (360.8 Wh kg^−1^),^15^ Zn//NaV_3_O_8_•1.5 H_2_O batteries (300 Wh kg^−1^),^35^ aqueous Na ion batteries (≈33 Wh kg^−1^),^44^, ^45^ and aqueous Li ion batteries (50−90 Wh kg^−1^).^46^, ^47^ In addition, our device has a higher energy density and power density compared to batteries assembled with other MnO_2_ configurations, such as Zn//α‐MnO_2_ batteries,^9^ Zn//β‐MnO_2_ batteries,^20^ Zn//γ‐MnO_2_ batteries^48^ and Zn//todorokite‐type MnO_2_ batteries.^49^ In addition to the gravimetric performance, the volumetric energy density and cycle stability of the quasi‐solid‐state ZMB device also show significant superiority, compared to those in the literature (Figure 5g). In detail, our ZMB exhibits a high volumetric energy density of 35.11 mWh cm^−3^, which is higher than many reported flexible energy storage devices including Zn–MnO_2_ batteries (34 mWh cm^−3^),^31^ Ni–Bi batteries (16.9 mWh cm^−3^),^43^ Ni–Zn batteries (7.76 mWh cm^−3^),^42^ Ni–Fe batteries (7.21 mWh cm^−3^),^50^ and even Li thin film batteries.^51^

Furthermore, the cycling time is among the best of the battery devices in Figure 5g. The cycling performance at 10 mA cm^−2^ up to 10 000 times for our quasi‐solid‐state device is further shown in Figure 5h, which indicates a very stable capacity retention as well as high coulombic efficiencies after long‐term charging and discharging. Such excellent cycling behavior has never been demonstrated in flexible ANZMBs using conventional gel electrolytes before.

Based on the above, our ZMB device can simultaneously exhibit high energy density, high power density and ultralong cycling stability. In addition to the benefits from the unique layered structure of δ‐MnO_2_, the integrated device architecture assembled with the adhesive gel electrolyte is believed to promote electrochemical stability.

**Figure 6:**
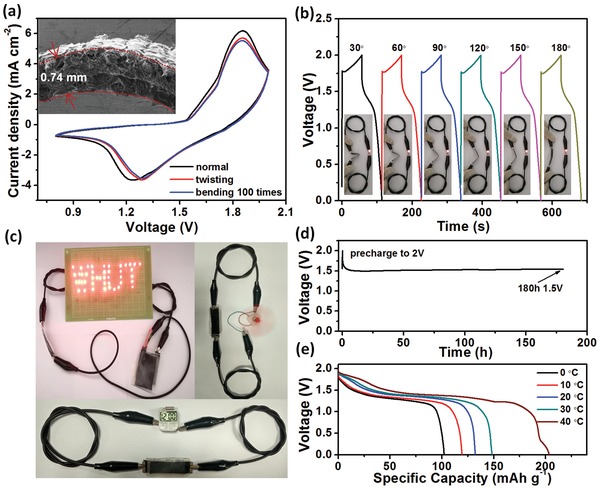
a) CVs of our flexible device with different shape deformations. Inset is the device SEM image in a bending state. b) GCD curves with different bending angles. Insets are optical photos of the device powering an LED light. c) LED pattern, rotating motor, and electronic watch driven by the charged flexible device. d) Self‐discharge curve. e) GCD curves at different temperatures at 5 mA cm^−2^.

Quasi‐solid‐state energy storage devices have a number of amazing advantages such as improved safety, easy packaging, excellent flexibility, and so on. To manifest the potential of our ZMB device for practical applications in portable and wearable electronic devices, the electrochemical performance was investigated by considering real conditions. **Figure** **6**a shows CV curves of the ZMB device after being subjected to different shape deformations. As demonstrated, the CVs almost overlap with the normal state, even when the device is twisted or bent 100 times, which is indicative of impressive robustness and high flexibility. A cross‐sectional SEM image of the bent device is shown in the inset of Figure 6a. No obvious cracks or detachments occur between the electrode and electrolyte, which further confirms the excellent robustness of the device under shape change. Such a good mechanical attribute should be strongly related to the integrated device structure, in which the gel electrolyte penetrates into the binder‐/additive‐free δ‐MnO_2_ array film.

Moreover, GCD profiles with various bending degrees were recorded and are shown in Figure 6b, demonstrating negligible changes in voltage plateaus and capacity (also see Figure S12, Supporting Information). The inserted optical images reveal that such bending of the devices can still power a light‐emitting diode (LED) brightly. Figure 6c further illustrates that 44 LEDs (3 mm diameter red; 1.8 V, 20 mA; designed with a pattern of “WHUT”), a mini‐electric fan and an electronic watch can all be efficiently powered by our flexible quasi‐solid‐state ZMBs. The self‐discharge curve (Figure 6d) of the device further reveals that after being charged to 2.0 V, the device quickly switches to a plateau voltage of ≈1.5 V and remains at the value for 180 h (≈100% voltage stability), showing great application potential with extremely low self‐discharging. For practical applications in flexible/wearable electronics, energy storage devices should have the ability to adapt to the ambient temperature changes. We thus performed GCD tests of our device at 5 mA cm^−2^ at different temperatures. In Figure 6e, it is obvious that the discharge capacity of the ZMB gradually decreases with temperature. Nevertheless, there is still ≈68.96% capacity (≈100 mAh g^−1^) remaining at 0 °C compared to that at 30 °C. In addition, even with a temperature increase to 40 °C, the device still works well with a good discharge plateau and capacity of 203.65 mAh g^−1^. The above results unambiguously reveal the good temperature adaptation of our flexible solid‐state ZMB.

**Figure 7:**
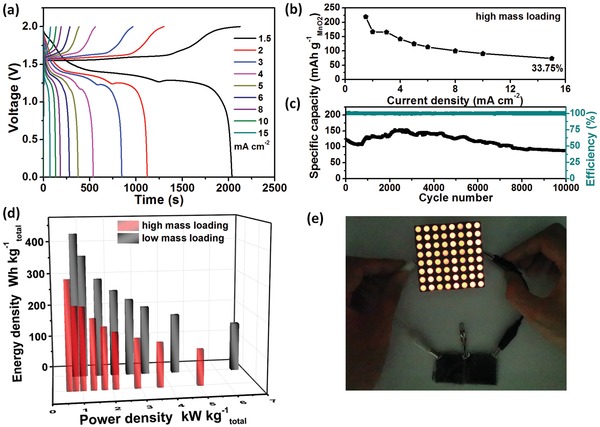
Electrochemical performance of the ANZMB device based on high δ‐MnO_2_ mass loading cathode. a) Charge–discharge profiles. b) Rate performance. c) Cycling performance at 10 mA cm^−2^. d) Ragone plot. e) A large 8 × 8 LED lattice board could be powered by the device.

Our binder‐free δ‐MnO_2_ cathode can be easily fabricated with high mass loading via extending the deposition time to achieve relatively high areal capacity. With the high mass loading of ≈5 mg cm^−2^, the flexible ANZMB still exhibits excellent charging/discharging plateaus with small polarization (**Figure** **7**a). From Figure 7b, the discharge capacity is estimated as ≈219.58 mAh g^−1^_MnO2_ at 1.5 mA cm^−2^, with the corresponding areal capacity of 1.1 mAh cm^−2^. In addition, the device shows good rate performance; 33.79% of the initial capacity can be maintained when the current density is increased tenfold from 1.5 to 15 mA cm^−2^. The long‐term cycling stability is also kept (Figure 7c). Although the electrochemical performance of the device is slightly inferior to that with low mass loading, the maximum energy and power densities can still reach 319.39 Wh kg^−1^ and 5.03 kW kg^−1^, respectively (Figure 7d). As further demonstrated in Figure 7e, two devices in series based on high mass loading electrode can easily power a large 8 × 8 LED (5 mm diameter red; 1.8 V, 20 mA) lattice board after fully charged.

## 3 Conclusions

In summary, we developed a high‐performance flexible ANZMB based on a δ‐MnO_2_ cathode. The layered δ‐MnO_2_ structure with a large interspacing for Zn^2+^ insertion is systematically investigated using the “noninterferencing” electrode platform. A “layered to layered” evolution mechanism is clarified, which is different from the majority of previously proposed mechanisms in other phased MnO_2_ electrodes characteristic of the 1st cycle phase transition. In our device, both electrodes are prepared on flexible current collectors via a simple electrodeposition method, which gives rise to a binder‐ and additive‐free architecture for fast ion diffusion and rapid electron transport. Benefiting from the specific crystal structure of δ‐MnO_2_ and electrode architectural merits, as well as an integrated device design, our ANZMB can simultaneously achieve high energy density, high power density and outstanding cycling stability up to 10 000 times, even with high mass loading. The robust flexibility of the device is demonstrated, with no obvious electrochemical performance degradation under serious bending and twisting of the device. Additionally, our device exhibits a low self‐discharge rate and can be operated within a broad temperature range, implying great potential in practical applications.

## 4 Experimental Section

*Synthesis of δ‐MnO_2_ Nanoflake Film Cathode*: The δ‐MnO_2_ cathode was synthesized using a rapid and mild electrodeposition method. Before electrodeposition, 0.01 m Mn(Ac)_2_ and 0.02 m NH_4_Ac were prepared as the electrodeposition solution; a CC current collector was cleaned with hydrochloric acid and ethanol using an ultrasonic cleaner for 15 min, was washed in distilled water three times, and was dried. The electrodeposition was performed in a typical three‐electrode setup on a CS 310 electrochemical workstation. In detail, the treated CC was utilized as the working electrode, and a Pt plate and a saturated calomel electrode (SCE) were used as the counter and reference electrodes, respectively. For growth, constant current polarization mode at 0.4 mA cm^−2^ was utilized for 10 min (0.5 mg cm^−2^; the loading mass could be readily controlled within 0.2–5.0 mg cm^−2^ by changing the polarization time). Finally, the as‐prepared sample was cleaned in deionized water several times to remove the residual electrolyte and was then annealed at 400 °C for 30 min in a muffle furnace to improve interfacial adhesion.

*Preparation of Zn Film Anode*: The Zn negative electrode was also synthesized using the electrodeposition method on CC. In detail, 0.2 m ZnSO_4_ mixed with 0.5 m trisodium citrate dehydrate (Na_3_C_6_H_5_O_7_·2H_2_O) electrolyte was first prepared. CC, Pt plate, and an SCE were utilized as the working, counter, and reference electrodes, respectively. Constant voltage polarization mode was utilized to electrodeposit a Zn metal film on CC at −1.4 V for 10 min (the loading mass was about 1.2 mg cm^−2^). After deposition, the CC was washed with distilled water several times and was dried in an oven at 60 °C for 4 h.

*Gel Electrolyte Preparation and Flexible ZMB Assembly*: To prepare the gel electrolyte, PVA (1799) powders were slowly added to deionized water and were stirred for 1 h at 80 °C to completely dissolve the powders. Then, 2 m ZnSO_4_ and 0.1 m MnSO_4_ mixed solution was added to the above viscous solution and was stirred for 1 h to form a sol electrolyte at room temperature. The weight ratio of PVA:ZnSO_4_ was 10:4. The flexible quasi‐solid‐state ZMB device was assembled with the δ‐MnO_2_ cathode, Zn anode, and gel electrolyte. Prior to assembly, electrodes were first infiltrated with the sol electrolyte and left under ambient conditions to remove the redundant water. Then, they were placed face to face and left until the electrolyte was gelled (semisolid). Finally, the device was packed with parafilm for further testing. The total thickness of the device was ≈0.74 mm.

*Materials Characterizations*: The morphologies and structures of the electrodes and device were studied by SEM (Hitachi S‐4800, Japan), HRTEM (JEM‐2010FEF, 200 kV), HAADF‐STEM (Titan Cubed Themis G2 300), Raman (Witech CRM 200) using 632.8 nm He–Ne laser, and XRD (Bruker D‐8 Advance) with Cu Kα irradiation (λ = 1.54 Å). The surface chemical composition of δ‐MnO_2_ was analyzed by XPS (Escalab 250‐Xi, USA). The mass of the electrode materials was measured on an AX/MX/UMX Balance (METTLER TOLEDO, maximum = 9.9 g, *d* = 0.001 mg).

Electrochemical measurements were performed on an electrochemical workstation (CS 310). To investigate the electrode properties, a three‐electrode system was employed, whereas for device testing, a two‐electrode mode was chosen. The electrolyte (if used) was 2 m ZnSO_4_ and 0.1 m MnSO_4_ aqueous solution. Electrochemical impedance spectroscopy (EIS) was measured from 0.01 Hz to 1 MHz at open circuit potential with a potential amplitude of 10 mV. GITT measurement was tested on LANHE battery testing system.

Specific capacities were calculated according to $C_{\text{s}}=\frac{\;\int_{0}^{\Delta t}I\times \text{d}t}{m}$, where *I*, Δ*t*, and *m* represent the discharge current, total discharge time, and the mass of active materials, respectively. Specific energy and power densities (*E* and *P*) of the ZMB device were obtained using the equations $E\;=\frac{\int IV(t)\text{d}t}{m}\;$ and $=\frac{E}{\Delta t}$, where *V*(t) is the discharging voltage at *t*, d*t* is time differential, Δ*t* is the discharge time, and *m* is the total mass of the active materials. Alternatively, the volumetric energy and power densities were estimated in a similar way by replacing *m* with the device volume *T* (including the cathode, anode, and electrolyte).

The diffusion coefficient of Zn^2+^ was calculated based on the equation $D_{\text{GITT}}=\;\frac{4}{\pi }{\left(\frac{m_{\text{B}}V_{\text{m}}}{M_{\text{B}}S^{\;}}\right)}^{2}{\left(\frac{\Delta E_{s}}{\tau (\text{d}E_{\tau }\text{/d}\sqrt{\tau })}\right)}^{2}\approx \frac{4}{\pi }{\left(\frac{{\text{m}}_{\text{B}}V_{\text{m}}}{M_{\text{B}}S}\right)}^{2}{\left(\frac{\Delta E_{\text{S}}}{\Delta E_{\tau }}\right)}^{2}\left(\tau \ll \frac{L^{2}}{D_{\text{GITT}}}\right)$, where *m*_B_ is the mass of the active material, *M*_B_ is the molecular weight (g mol^−1^) and *V*_m_ is its molar volume (cm^3^ mol^−1^), *S* is the total contacting area of electrode with electrolyte, τ is the duration time of the current pulse, Δ*E*_s_ is the difference in the open circuit voltage measured at the end of the relaxation period for two successive steps, $\text{d}E_{\tau }\text{/d}\sqrt{\tau }$ is the slope of the linearized region of the potential *E*_τ_ during the current pulse of duration time τ, and *L* is the thickness of the electrode.

*Theoretical Calculation*: All ab initio calculations were based on the framework of the density functional theory (DFT) using the Vienna Ab Initio Simulation Package (VASP). The electron–ion interaction was described by the projector augmented wave (PAW) method. The structural stabilities of layered δ‐MnO_2_ were studied by the Perdew–Burke–Ernzerhof (PBE) exchange‐correlation (XC) functions. For the geometric relaxation of the structures, summation over the Brillouin Zone (BZ) was performed with Monkhorst‐Pack *k*‐point intervals limited below 0.04 Å^−1^. A plane‐wave energy cutoff of 600 eV was used in all calculations. All structures were geometrically relaxed until the total force on each ion was reduced below 0.01 eV Å^−1^. The diffusion coefficient was related to the average mean square displacement (MSD) from molecular dynamics runs at each temperature *T* over a period of time (*t*),〈[Δ*r*(*t*)]^2^〉, as $D\;=\frac{〈{\left[\Delta r(t)\right]}^{2}〉}{2dt}$, where *d* is the dimensionality factor (*d* = 2 for a 2D system). AIMD run lasted for 80 ps after a 10 ps pre‐equilibrium run, meanwhile a time step of 2 fs in the *NVT* ensembles together with a Nosé–Hoover thermostat was employed.

## Conflict of Interest

The authors declare no conflict of interest.

## Supporting information

Supporting Information
