# Supplementary material for: Noninterference Revealing of “Layered to Layered” Zinc Storage Mechanism of δ‐MnO2 toward Neutral Zn–Mn Batteries with Superior Performance
Source: Adv Sci (Weinh). 2020 Jan 16;7(6):1902795. doi: 10.1002/advs.201902795 (PMC7080538; doi:10.1002/advs.201902795)
Supplement: Supplementary file 1 — Supporting Information [file ADVS-7-1902795-s001.pdf]

Copyright WILEY-VCH Verlag GmbH & Co. KGaA, 69469 Weinheim, Germany, 2020.

ADVANCED SCIENCE
Open Access

Supporting Information

for *Adv. Sci.,* DOI: 10.1002/advs.201902795

Noninterference Revealing of "Layered to Layered" Zinc Storage Mechanism of δ-MnO_2 toward Neutral Zn–Mn Batteries with Superior Performance

*Yuqi Jiang, Deliang Ba, Yuanyuan Li, and Jinping Liu**

Copyright WILEY-VCH Verlag GmbH & Co. KGaA, 69469 Weinheim, Germany, 2018.

Supporting Information

for *Advanced Science*, DOI: 10.1002/((please add manuscript number))

Non-Interference Revealing of “Layered to Layered” Zinc Storage Mechanism of δ-MnO_2 towards Neutral Zn-Mn Batteries with Superior Performance

*Yuqi Jiang, Deliang Ba, Yuanyuan Li, and Jinping Liu**

Y. Q. Jiang, Prof. J. P. Liu
State Key Laboratory of Advanced Technology for Materials Synthesis and Processing and School of Chemistry, Chemical Engineering and Life Science
Wuhan University of Technology
Wuhan, Hubei 430070, P. R. China
E-mail: liujp@whut.edu.cn

D. L. Ba, Prof. Y. Y. Li
School of Optical and Electronic Information
Huazhong University of Science and Technology
Wuhan 430074, P. R. China

Prof. J. P. Liu
State Center for International Cooperation on Designer Low-carbon & Environmental Materials and School of Materials Science and Engineering
Zhengzhou University
Zhengzhou 450001, Henan, P. R. China

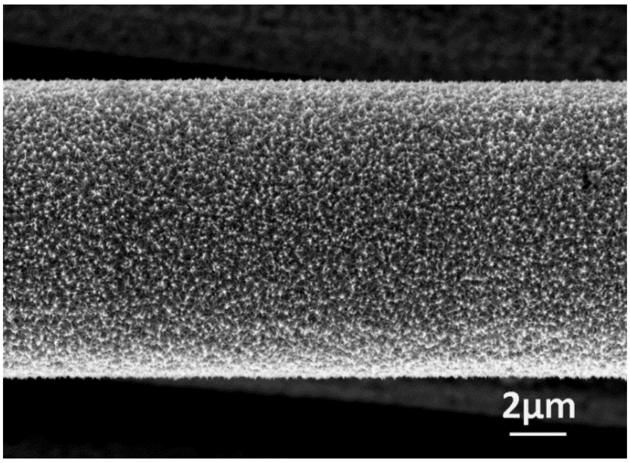

Figure S1. Low-magnification SEM of δ-MnO_2 cathode.

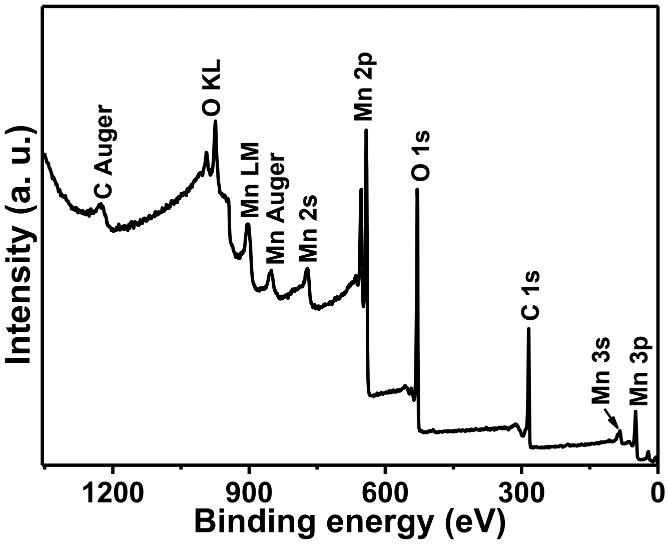

Figure S2. Full XPS spectrum of δ-MnO_2.

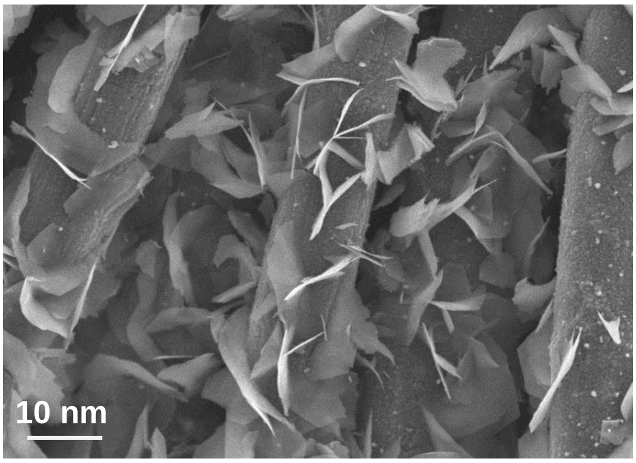

Figure S3. SEM of δ-MnO_2 cathode at state 2.

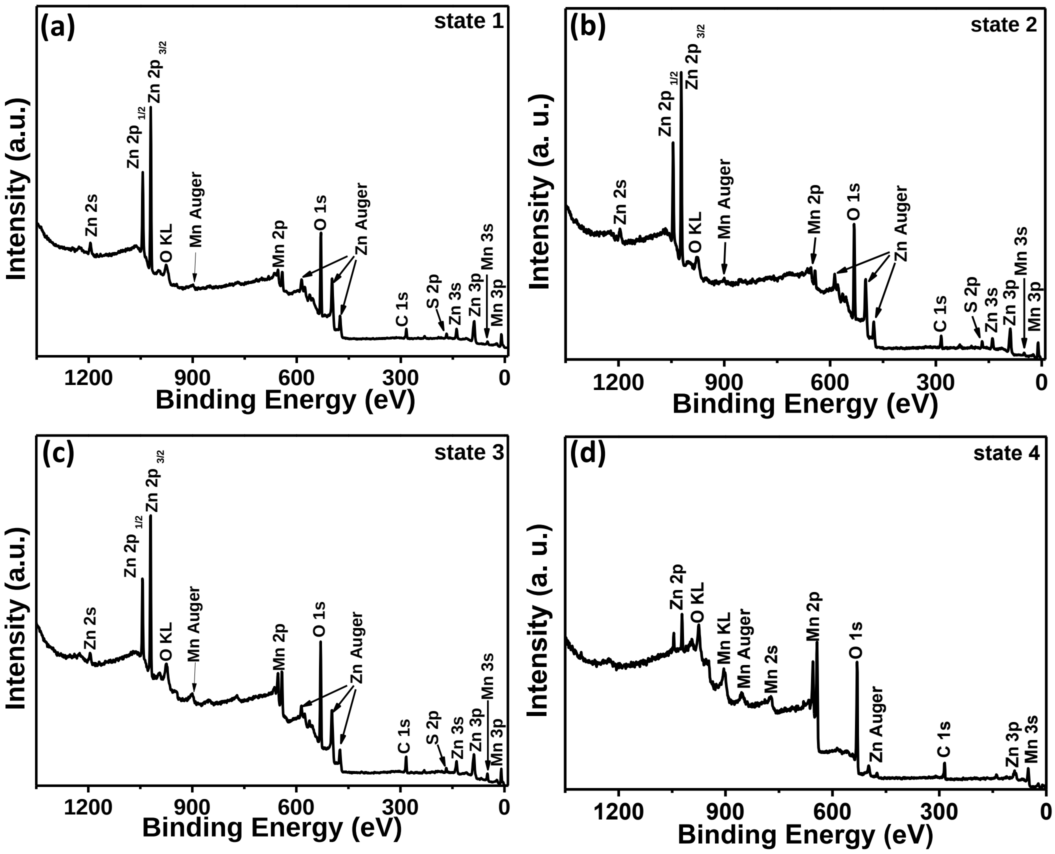

Figure S4. XPS of (a) state 1, (b) state 2, (c) state 3 and (d) state 4 of δ-MnO_2 cathode.

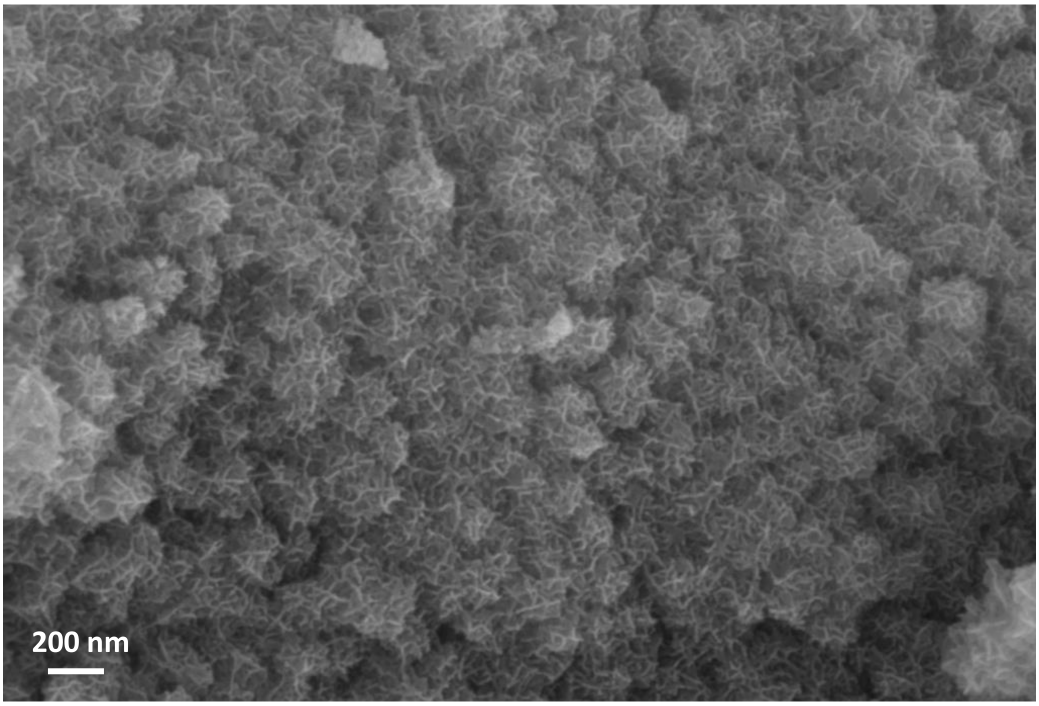

Figure S5. SEM image of δ-MnO_2 cathode at state 4 after 10000 cycles.

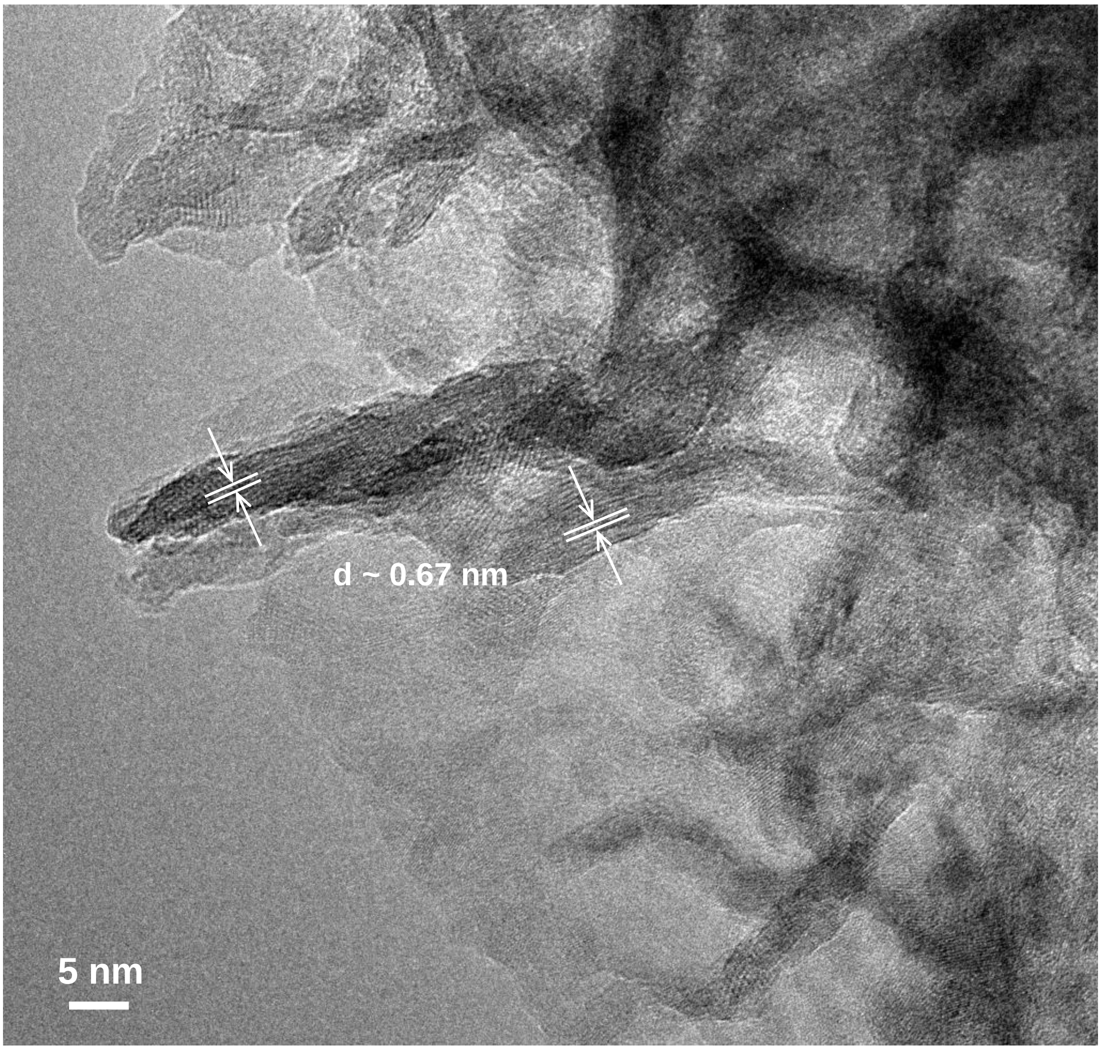

Figure S6. HRTEM image of δ-MnO_2 cathode at state 4 after 10000 cycles.

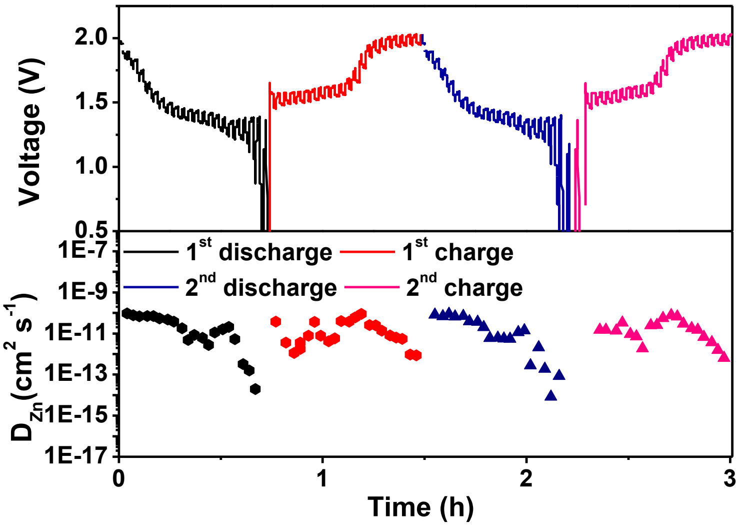

Figure S7. GITT profiles and calculated Zn^{2+} diffusion coefficients of δ-MnO_2 cathode.

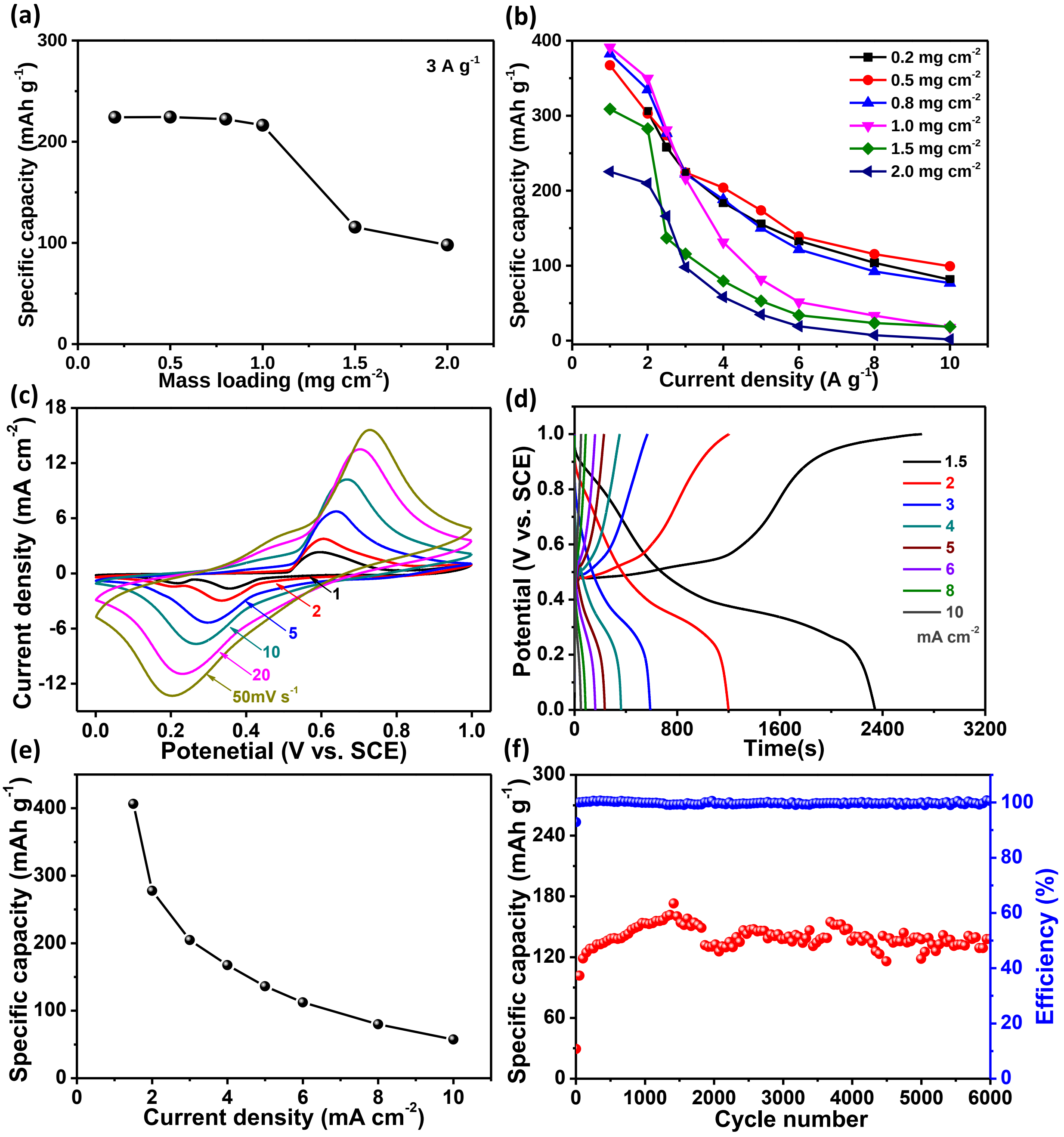

Figure S8. Electrochemical performance of δ-MnO_2 electrode in three-electrode system. (a) Plot of specific capacity versus loading mass at 3 A g^{-1}. (b) Rate performance comparison for different loading mass. (c) CV curves at various scan rates. (d) the charge-discharge curves. (e) rate performance. (f) cycling performance at 5 mA cm^{-2} and Coulombic efficiency.

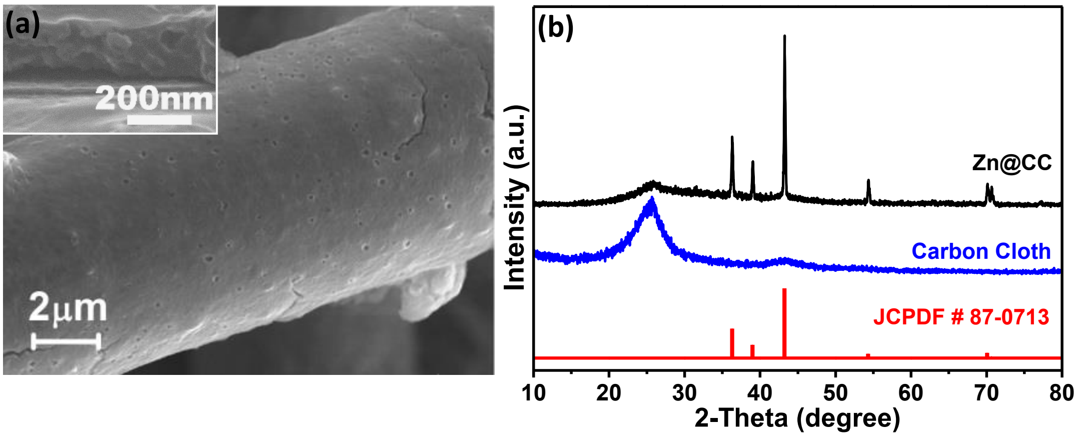

Figure S9. Structural and morphological characterizations of the Zn anode: (a) SEM images; (b) XRD pattern.

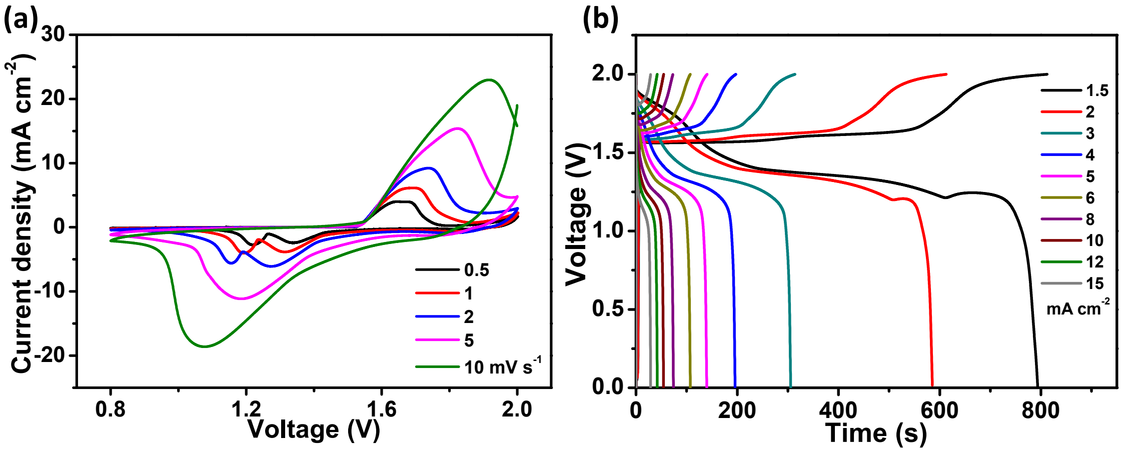

Figure S10. Electrochemical performance of the ANZMB. (a) CVs and (b) GCD curves in aqueous electrolyte.

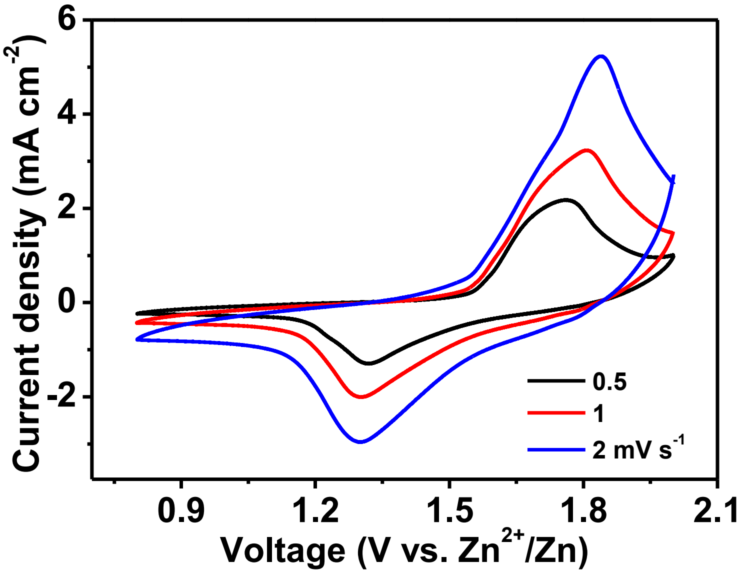

Figure S11. CV curves of the flexible ZMB device.

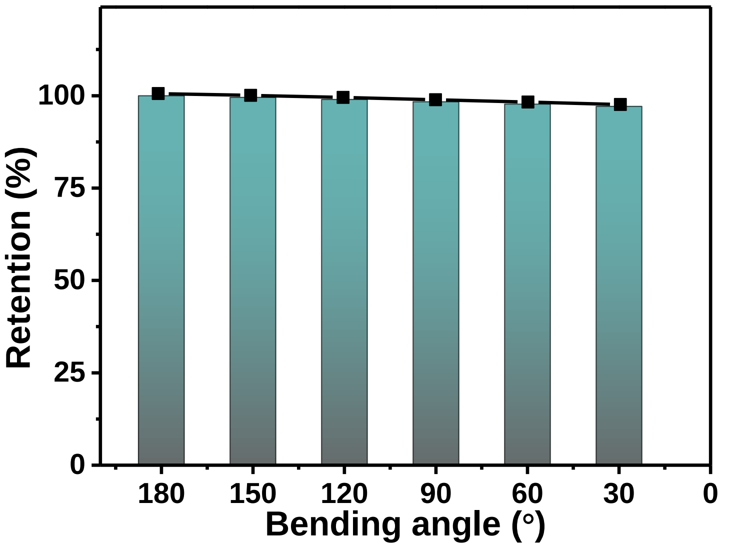

Figure S12. Capacity retention ratios of the flexible device with different bending angles.
